# Hepatoprotective and Renoprotective Properties of Lovastatin-Loaded Ginger and Garlic Oil Nanoemulsomes: Insights into Serum Biological Parameters

**DOI:** 10.3390/medicina55090579

**Published:** 2019-09-09

**Authors:** Syed Ali Faran, Sajid Asghar, Syed Haroon Khalid, Ikram Ullah Khan, Muhammad Asif, Ikrima Khalid, Umar Farooq Gohar, Tanveer Hussain

**Affiliations:** 1 Faculty of Pharmaceutical Sciences, Government College University Faisalabad, Faisalabad 38000, Pakistan; 2 Institute of Industrial Biotechnology, Government College University Lahore, Lahore 54000, Pakistan; 3 Faculty of Engineering and Technology, National Textile University, Faisalabad 38000, Pakistan

**Keywords:** lovastatin, anti-hyperlipidemia, hepatoprotective, renoprotective, ginger oil, garlic oil, nanoemulsomes

## Abstract

*Background and Objectives*: Dyslipidemia is gaining much attention among healthcare professionals because of its high association with the malfunctioning of a number of normal physiological and metabolic processes in the body. Obesity is directly interconnected with dyslipidemia and is said to be a denouement of hyperlipidemia and, if left untreated, may lead to intense damage to organs that are directly involved in fat metabolism. The objective of this study was to investigate the synergistic antiobesity and anti-hyperlipidemic activities along with hepato- and renoprotective potential of nanoemulsomes (NES) of lovastatin (LTN)-loaded ginger (GR) and garlic (GL) oils. *Materials and Methods*: LTN nanoemulsomes co-encapsulated with GR oil and GL oil were prepared by a thin hydration technique. Eight-week-old male Wistar rats weighing 200–250 g were induced with hyperlipidemia via a high-fat diet (HFD) comprising 40% beef tallow. Body weight, serum biochemical lipid parameters, and those for liver and kidney functions, serum TC, LDL-C, vLDL-C, HDL-C, TG, atherogenic index (AI), ALT, AFT, ALP, γ-GT, total protein (TP), serum albumin and globulin ratio (A/G), serum creatinine, blood urea nitrogen (BUN) and blood urea, and histopathology of hematoxylin and eosin (H&E) stained liver and kidney sections of all aforementioned groups were examined in the treated animals. *Results*: Nanoemulsomes of LTN-loaded GR and GL oils provided synergistic effects with LTN, exerted better ameliorative actions in reducing serum TC, LDL-C, vLDL-C, triglycerides, and AI, and improved serum HDL-C levels. Serum ALT, AST, ALP, and γ-GT levels were in the normal range for nanoemulsome groups. H&E stained liver and kidney sections of these animals confirmed better hepatoprotective and renoprotective effects than LTN alone. Serum biochemical parameters for renal functions also claimed to be in the moderate range for nanoemulsome-treated groups. *Conclusion*: This study demonstrated that nanoemulsomes of LTN-loaded GR and GL oils synergistically provided better antihyperlipidemic, hepatoprotective, and renoprotective effects as compared to LTN alone.

## 1. Introduction

Dyslipidemia is known to be a metabolic disorder of lipid metabolism that distends as a result of inappropriate lipid metabolism (lipogenesis and lipolysis), ultimately leading to obesity and other diseases [1]. Obesity, in the modern world, is dramatically challenging our confidence, originating from the drastically changing lifestyle of the general population [2]. Obesity, along with hyperlipidemia and hypertension, is considered to be highly interconnected with the swift instigation of nonalcoholic fatty liver disease (NAFLD) [3]. NAFLD prevails more in obese patients than nonobese ones. Its prevalence is up to 30%–60% in obese adults, and about 20%–25% of this population is prone to nonalcoholic steatohepatitis (NASH) or may also develop end-stage hepatic cancer if left untreated [4]. NAFLD is considered to occur from the upregulation of enzymes including HMG Co-A reductase, sterol regulatory element-binding protein-1c (SREBP1-c), and acetyl coenzyme A carboxylase (ACC), which are directly involved in cholesterol synthesis. These enzymes are triggered upon excess deposition of saturated and free fatty acids (FFA) in adipocytes, owing to intake of a fat-rich diet [5]. 

The relationship between renal injury and excessive lipid intake was found one and a half centuries ago by Virchow in 1858, who claimed that renal epithelium fatty degeneration occurred in Bright’s disease [6]. Later, a number of investigators discovered that epithelial cell injury and mesangial proliferation caused by lipids have direct roles in the progression of renal diseases [7]. High fat intake has direct involvement in the advent of cardiovascular, hepatic, and renal abnormalities. Moreover, dyslipidemia and/or hyperlipidemia induced by a high-fat diet are known to aggressively affect the normal physiology of kidneys. Among various hyperlipidemia-induced renal abnormalities, glomerulosclerosis and lipid-induced oxidative stress are two major problems that ultimately lead to altered or abnormal functioning of nephrons, and they may progress to kidney failure or renal impairment if left untreated [8,9,10]. Podocyte injury and mesangial sclerosis are highly associated with elevated levels of plasma cholesterol and triglycerides [11]. High-density lipoprotein cholesterol (HDL-C) and low-density lipoprotein cholesterol (LDL-C) generate high oxidative stress in renal injury, and this leads to lower lipoprotein production and worsens renal injury [6].

Hepatic and renal injuries, induced by excess lipid deposition in the body, can be reversed by a number of mechanisms and by using a number of naturally occurring therapeutic agents. Ginger (GR) and garlic (GL) are endowed with a long list of therapeutically active compounds that have shown promising results in compensating such aforementioned abnormalities [12,13]. GR contains various potent chemical constituents that are known to produce beneficial effects in normalizing a number of physiological functions [14]. GR, owing to its rich phytochemical history, has been shown to ameliorate total lipid profile of animals by imparting hepatoprotective and renoprotective effects in obesity-induced hyperlipidemic conditions in rat models [15,16,17,18].

Like GR, GL is also known for its remediation properties against fat-induced hepatic and renal injuries. GL contains a large proportion of organosulfur compounds such as allicin, S-allaylcystein, allyl sulfide, diallayl sulfide (DAS), and diallyl disulfide (DADS), which play pivotal roles in managing lipid-induced morphological changes to key organs. These compounds are proven to have antioxidant [19] and antihyperlipidemic [20] activities along with a specificity to provide renoprotective [21] and hepatoprotective effects [22].

Statins have been widely described as direct lipid-lowering agents. Besides this remarkable action, they have also been reported to exert protective actions to organs directly affected by the accumulation of fatty acids [23,24,25]. Lovastatin (LTN), a potent prodrug of the statin family, is the fermented product of red yeast rice [26]. The metabolic product of LTN tends to inhibit a rate-limiting enzyme, 3-hydroxy-3-methyl glutaryl coenzyme A (HMG Co-A) reductase, involved in the biosynthetic and metabolic pathways of cholesterol [27]. LTN has a direct role in lowering total cholesterol (TC) and LDL-C, hence preventing risks of coronary heart diseases [28]. Besides providing lipid-alleviating and hepatoprotective effects, LTN has also been widely reported to directly deliver renoprotective effects when the organ is majorly damaged due to high fat deposition [29].

All these aforementioned agents have been used alone or in combination with other xenobiotics. In this study, the antihyperlipidemic capacities of drug-loaded GR (Moon scientific traders, Punjab, Pakistan) and GL oil- nanoemulsomes (Moon scientific traders, Punjab, Pakistan) were measured with an emphasis on evaluating the synergistic effects of the active drug and oils on lipid metabolism. Serum biochemical analyses of various biomarkers of hepatic and renal functions were performed to establish the efficacy of drug-loaded GR and GL oil- nanoemulsomes.

## 2. Materials and Methods

LTN was obtained as a kind gift from Nabiqasim Industries, Pakistan. Stearic acid was cordially gifted by Saffron Pharmaceuticals Pvt. Ltd. Punjab, Pakistan. GR and GL oils were procured from Moon scientific traders, Punjab, Pakistan. Phospholipon 90G (lecithin stabilized with 0.1% ascorbyl palmitate) was a kind present from Lipoid AG, Steinhausen, Switzerland. Ethanol, chloroform, formaldehyde, and glycerol were procured from Musaji Adam & Sons, Karachi, Pakistan. All other solvents, chemicals, and reagents used were of analytical grade.

### 2.1. Preparation of Lipid Nanoformulations

Lipoidal nanovesicular systems (nanoemulsomes) were fabricated by the thin layer hydration technique with slight modifications [30]. Firstly, the lipid phase (0.1 g) was formed in 10 mL of an ethanol/chloroform (1:1 v/v) mixture at a 1:2 ratio of solid lipid to liquid lipid for both GR and GL oils, separately. Fixed amounts of drug and phospholipon 90 G (stabilizer) were added to lipid mixture (Table 1). The mixture of lipids was then dried in a rotary evaporator (RE-100 Pro, D-Lab, USA) maintained at 40 °C and 60 rpm, leaving behind a thin layer of residues on the walls of a round-bottom flask. This layer was hydrated in parts with 20 mL of a 10% v/v glycerol solution with continuous shaking for 15–20 min. Finally, this pre-emulsion was subjected to sonication using a probe-type ultrasonicator (3000 MP, BioLogics, Inc, Manassas, VA, USA) at 150 W for 5 min to yield nanoemulsomes. These formed nanoemulsomes were then stored at 4 °C until utilization.

### 2.2. Particle Size, Zeta Potential, and Polydispersity Index (PDI)

Particle size, zeta potential, and the polydispersity index (PDI) of nanoformulation suspensions diluted in double-distilled water were evaluated by dynamic light scattering (DLS) using Malvern Nano ZS90, UK. Results were obtained in triplicate, and their averages were taken.

### 2.3. Experimental Animals

Thirty male Wistar rats (8 weeks old) weighing 200–250 g were used in this hypercholesterolemic rat model. These rats were obtained from the Animal Laboratory, Faculty of Pharmaceutical Sciences, Government College University Faisalabad, Pakistan. All animals were kept in a well cleaned and ventilated animal room at a controlled room temperature of 25 ± 2 °C with a humidity level of 65% ± 5% over 12 h dark/light cycles with free access to basal diet and water ad libitum. Rats were acclimatized in this environment for one week before commencement of the experiment. Animal experimentation was carried out as per institutional and international guidelines for animal care and ethics and was approved by the Institutional Review Board, Government College University Faisalabad (Ref No. GCUF/ERC/2010, Study No. 19610, IRB No. 610, Dated 25 March 2019).

### 2.4. Experiment Protocol

Initially, for a period of 4 weeks, rats were divided into 2 groups: group I or group A (*n* = 6) was fed a normal control diet or basal diet, and group II (*n* = 24) was fed a high-fat diet containing 40% beef tallow [31]. At the beginning of the 5th week (initiation of treatment period), rats from group II were further randomly divided into 4 groups each comprising 6 rats (*n* = 6) and given treatments described below until the end of the 8th week.

Groupe A: Rats were given a normal control diet over a continuous period of 8 weeks.
Groupe B: Rats were fed only a high-fat diet and termed as the high-fat diet (HFD) group.
Groupe C: Rats were given pure LTN (dose = 30 mg/kg/d), suspended in 1.8% carboxymethyl cellulose (CMC) solution with concomitant administration of HFD.
Groupe D: Rats were kept on HFD along with administration of GR oil nanoemulsomes at an equivalent dose of 30 mg/kg/d.
Groupe E: Rats were kept on HFD along with administration of GL oil nanoemulsomes at an equivalent dose of 30 mg/kg/d.

During this period of 8 weeks, weights of all animals were noted on weekly basis to evaluate the effects of treatments on body weight as well. Antihyperlipidemic treatment was started at the end of the 4th study week and continued until the end of the 8th week of study.

### 2.5. Serum Biochemical Analyses for Evaluation of the Lipid Profile

Blood samples were taken from sacrificed animals in glass vacutainers and were immediately subjected to centrifugation at 6000 rpm for 15 min. The supernatant serum layer was then separated from each tube and was carefully stored at −20 °C until further analyses. Total cholesterol (TC), triglycerides (TG), low-density lipoproteins (LDL-C), very low density lipoproteins (vLDL-C), and high-density lipoproteins (HDL-C) were measured using commercially available diagnostic kits (Crescent diagnostics, Jeddah, Saudi Arabia) adopting the calorimetric method (CLARIOstar^®^, BMG LABTECH, SpectraMax^®^ M5e Microplate Reader). Atherosclerosis risk factor was calculated by computing atherogenic indices (AI) as per the following formula given in literature [32]. Furthermore, the TG/HDL-C ratio was investigated to calculate insulin resistance as reported elsewhere [33].

(1) $$AI=\frac{LDL+vLDL}{HDL}.$$

### 2.6. Serum Biochemical Analyses for Evaluation of the Liver Function Tests (LFTs)

Serum analyses for various biomarkers of LFTs were done using commercial kits (Crescent diagnostics, Jeddah). These biomarkers include alanine aminotransferase (ALT), aspartate aminotransferase (AST), gamma glutamyl transpeptidase (γ-GT), and alkaline phosphatase (ALP). Results were obtained following the colorimetric method. Data values were presented in international units per litter (IU/L).

### 2.7. Serum Biochemical Analyses for Evaluation of the Renal Function Tests (RFTs)

Various renal biomarkers such as serum levels of urea, creatinine, BUN, total protein, albumin, globulin, and the albumin/globulin ratio (A/G) were thoroughly examined to assess functioning of the kidneys in the presence of HFD and to evaluate the effect of GR and GL oils in synergy with LTN [34,35]. These results were presented as mg/dL or g/dL.

### 2.8. Histopathological Examination

Liver and kidneys from experimental animals were excised and were fixed in 10% buffered formalin solution. Later, fixed organs were embedded in paraffin, and sections of 5 µm thickness were prepared from each organ sample using a microtome. Later, tissue sections were stained with hematoxylin and eosin (H&E) and examined microscopically (ACCU-SCOPE 3000, Commack, NY, USA) for any pathological changes induced by a high-fat diet under supervision of pathologist, and images were captured via CaptaVision software (ACCU-SCOPE, Commack, NY, USA).

### 2.9. Statistical Analysis:

Data obtained were presented as mean ± SD. All data were subjected to one-way ANOVA with a post hoc Tukey test for multiple column comparisons using Graphpad Prism software. A *p* value less than 0.05 was considered significant.

## 3. Results

### 3.1. Preparation and Characterization of Nanoemulsomes

Both GR and GL oil- formulations formed by thin layer hydration showed uniform and homogeneous particles with average particle sizes of 240 and 120 nm (Table 2), respectively. These formulations indicated good stability, as their average zeta potential values were −24 and −20 mV, respectively. Particles in both dispersions displayed uniformity, as their PDI values were below 0.3. GL oil formulations were monodisperse and presented a much smaller size than the GR oil counterpart. The size of the formulation could play an important role in the performance of the nanopreparations.

### 3.2. Body Weight of Animals

**Figure 1:**
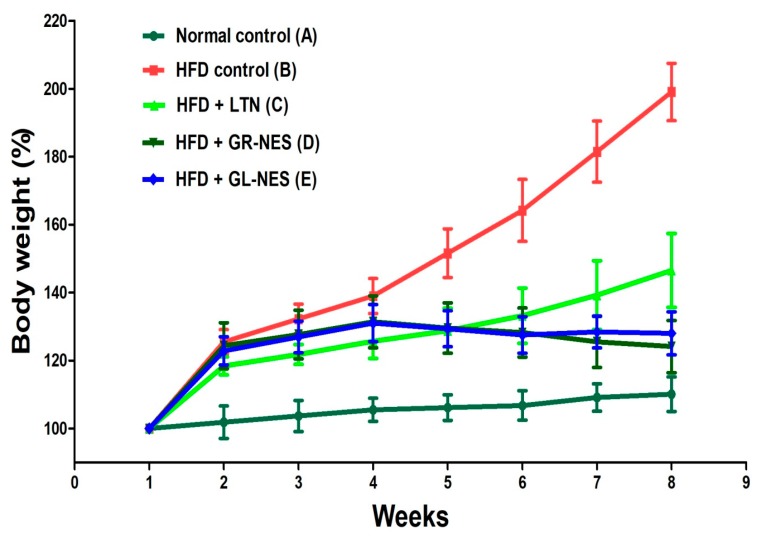
Percent body weight change of hyperlipidemic rat models (*n* = 6) for nanoformulations and pure LTN after 4 weeks of oral administration (dose = 30 mg/kg/d). HFD, high-fat diet.

Since animals in the normal control group (group A) were kept on a normal diet, they did not achieve any significant gain (*p* > 0.05) in their body weights throughout the experiment. From Figure 1, it can be clearly seen that percent body weight of all animals of the HFD group (groups B–E) significantly increased as compared to normal control group A over the first 4 weeks. By 4 weeks, animals from groups B–E achieved marked increases in their body weights at 138.9%, 125.6%, 130.9%, and 131.4%, respectively. Whereas, groups D and E displayed a slight loss in weight from the 5th to 8th week (treatment period). These results were better than group C (group treated with pure LTN suspension), which showed a slightly uplifted weight curve during the treatment period. After the 6th week, the percent weight of group D further decreased, while that of group E achieved a sustained level. At the completion of 8 weeks, the total body weight of animals of group B increased up to 200%, while that of group C increased 146%. Groups D and E showed a slight decline (i.e., 7% and 3%, respectively) as compared to the weight percent of the initial 4 weeks.

### 3.3. Lipid Profile

#### 3.3.1. Total Cholesterol (TC)

**Figure 2:**
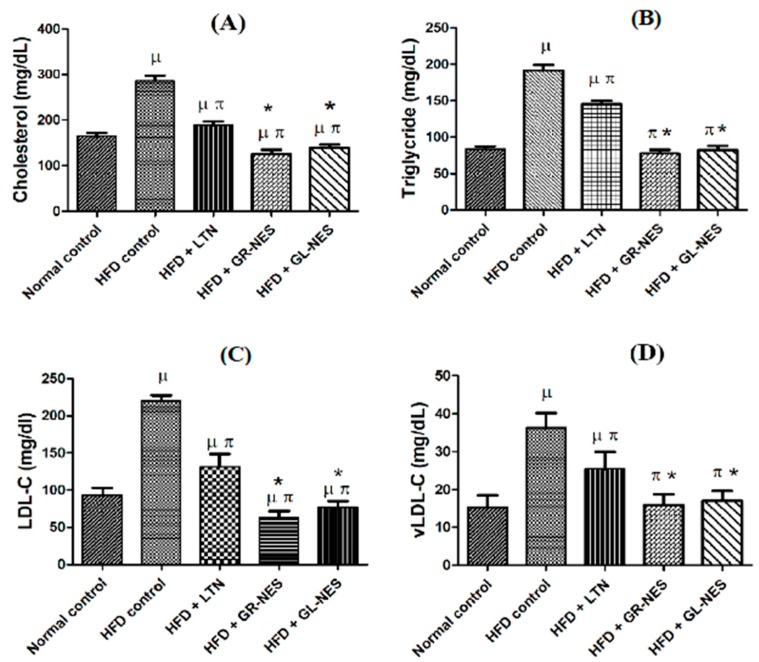
(**A**) Serum total cholesterol (TC); (**B**) Serum triglycerides (TG); (**C**) Serum low-density lipoprotein cholesterol (LDL-C); (**D**) Serum very low density lipoprotein cholesterol (vLDL-C) of nanoformulations and pure LTN in hyperlipidemic rat models (*n* = 6) after 4 weeks of oral administration (dose = 30 mg/kg/d). µ indicates *p* < 0.05 vs. normal control; π indicates *p* < 0.05 vs. HFD control; * indicates *p* < 0.05 vs. HFD + LTN.

The TC level of group B significantly increased (*p* < 0.05) by 175% (280 ± 11.68 mg/dL) over a period of 8 weeks as compared to that of group A (160 ± 7.23 mg/dL; Figure 2A). Whereas, in comparison to group A, TC levels of rats of groups C–E were 115% (184 ± 7.19 mg/dL), 73% (117 ± 9.24 mg/dL), and 82% (131 ± 7.4 mg/dL), respectively. While amongst treated groups, there was a decrease of 37% and 29% in serum TC levels of groups D and E, respectively, as compared to that of the pure LTN-treated group (*p* < 0.05). This shows substantial lowering of serum TC levels in groups of drug-loaded nanoemulsomes.

#### 3.3.2. Triglycerides (TG)

The TG level of the high-fat diet group B over a period of 8 weeks was the highest at 192 ± 7.4 mg/dL (231% more than that of normal control group A). The group treated with LTN suspension in 1.8% CMC solution showed a 25% (144 ± 4.34 mg/dL) lower TG level as compared to group B (Figure 2B). While groups D and E displayed significant reductions (*p* < 0.05) in TG levels as compared to group B (i.e., 77.5 ± 4.84 mg/dL (40% lower than group B) and 81 ± 6.16 (42% lower than group B), respectively). When comparing percentage increase or decrease in TG levels of all treated animals with that of the normal control diet group A, groups D and E showed significant reductions (*p* < 0.05) of 3.4% and 8.2% reduced TG levels, respectively.

#### 3.3.3. Low-Density Lipoprotein Cholesterol (LDL-C)

After the experiment was terminated, HFD control group B showed a 231% (210 ± 6.95 mg/dL) increase in LDL-C level against normal control group A (90.6 ± 9.02 mg/dL). Groups D and E showed significantly (*p* < 0.05) reduced LDL-C levels: 71.8 ± 7.21 mg/dL (29% lower than group A) and 86.5 ± 7.88 mg/dL (5.1% lower than group A). Pure LTN suspension in CMC solution did not significantly reduce LDL-C levels (163.9 ± 6.64 mg/dL) when compared with those of control diet group A (*p* > 0.05). Instead, it showed a higher LDL-C level when compared to groups treated with GR and GL oil (Figure 2C) drug-loaded lipid nanoformulations (*p* < 0.05).

#### 3.3.4. Very Low Density Lipoprotein Cholesterol (vLDL-C)

vLDL-C levels of all groups were compared with those of normal diet group A (Figure 2D). Drug-loaded GR and GL oil nanoemulsomes ameliorated the vLDL-C profile of rats in a better way as compared to pure LTN CMC suspension (group C). vLDL-C levels for these groups were 15.4 ± 2.84 mg/dL (53% lower than group C) and 16.2 ± 2.7 mg/dL (56% lower than group C), respectively. In comparison to the normal diet group whose vLDL-C level was 16.6 ± 3.07 mg/dL, there were respective 3% and 8% decreases in vLDL-C levels of groups administered with GR and GL oil nanoformulations. The pure LTN suspended solution showed 28.8 ± 4.54 mg/dL vLDL-C levels, which was 173% greater than those of normal control group A.

#### 3.3.5. High-Density Lipoprotein Cholesterol (HDL-C)

**Figure 3:**
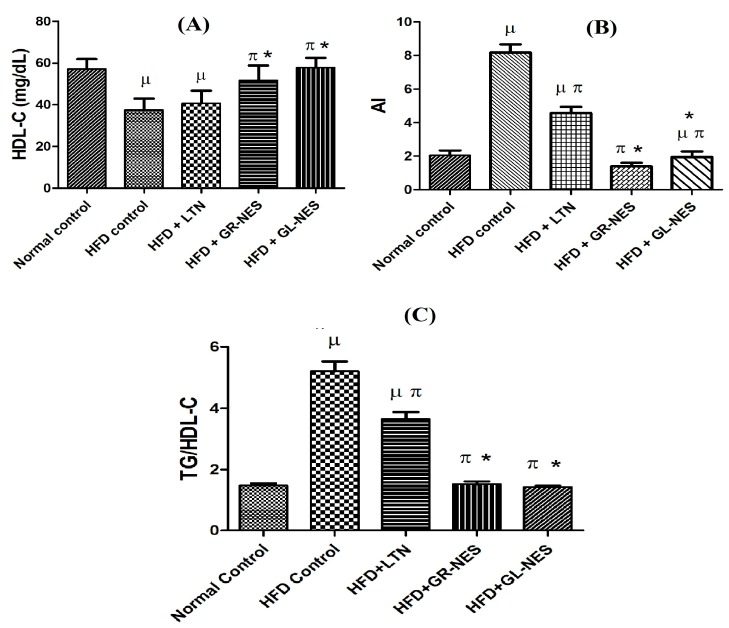
(**A**) Serum high-density lipoprotein cholesterol (HDL-C); (**B**) atherogenic index (AI); (**C**) TG/HDL-C to indicate insulin resistance of nanoformulations and pure LTN in hyperlipidemic rat models (*n* = 6) after 4 weeks of oral administration (dose = 30 mg/kg/d). µ indicates *p* < 0.05 vs. normal control; π indicates *p* < 0.05 vs. HFD control; * indicates *p* < 0.05 vs. HFD + LTN.

Normal control group A showed 52.8 ± 4.72 mg/dL of HDL-C after completion of the animal experiment. Groups D and E showed quite satisfactory results in improving HDL-C levels of rats (Figure 3A). There was no significant difference (*p* > 0.05) between HDL-C levels of these groups and normal control group A: HDL-C levels were 49.8 ± 7.17 mg/dL (6% of group A) and 52.3 ± 4.62 mg/dL (1% of group A), respectively. HFD control group B showed a significant reduction (*p* < 0.05) in HDL-C levels (i.e., 58% (31 ± 5.51 mg/dL) lower than group A). The HDL-C level of rats treated with pure LTN suspension was 41.3 ± 6.13 mg/dL. This illustrates respective 17% and 21% lower levels than those of group D and E, indicating that nanoformulations administered to these groups improved HDL-C levels in a better way as compared to the simple LTN suspension.

#### 3.3.6. Atherogenic Index (AI) and Insulin Resistance

Atherosclerosis occurrence was the lowest among animals treated with LTN nanoemulsomes. Results were compared with AI of control group A, and there was no significant difference (*p* > 0.05) between groups A and E regarding AI, while groups A and D were significantly different (*p* < 0.05). On the other hand, hyperlipidemic rats (group B) and those treated with pure LTN suspension (group C) showed significant differences against group A. Similarly, both groups D and E showed significant reduction in AI as compared to group C (*p* < 0.05) and further decreased the risk of atherosclerosis (Figure 3B). These results illustrate that GR and GL oil nanoemulsomes helped to combat lipid-induced fatalities by improving the lipid profile even after concomitant intake of a fat-rich diet. 

The TG to HDL-C ratio (TG/HDL-C) has been suggested as a surrogate marker for detection of insulin resistance [33]. A TG:HDL-C ratio of ≥3 has been shown to be closely correlated to insulin resistance. In the clinical scenario, the TG/HDL ratio has been proven to have a high correlation with the prevalence of metabolic syndrome with insulin resistance [36]. Figure 3C shows the TG/HDL to indicate insulin resistance in the animals of treatment groups. The HFD group developed the worst insulin resistance, as the TG/HDL-C ratio was greater than 5. LTN treatment alone was not sufficient to avoid insulin resistance in the rats, as the TG/HDL-C ratio was around 3.5. Nonetheless, GR-NES and GL-NES were able to reverse insulin resistance in the rats. The TG/HDL-C ratios of normal rats and nanoemulsome-treated rats were quite similar (*p* > 0.05), and the values did not exceed 1.6.

### 3.4. Liver Function Analyses (LFTs)

#### 3.4.1. Alanine Aminotransferase (ALT or SGPT)

**Figure 4:**
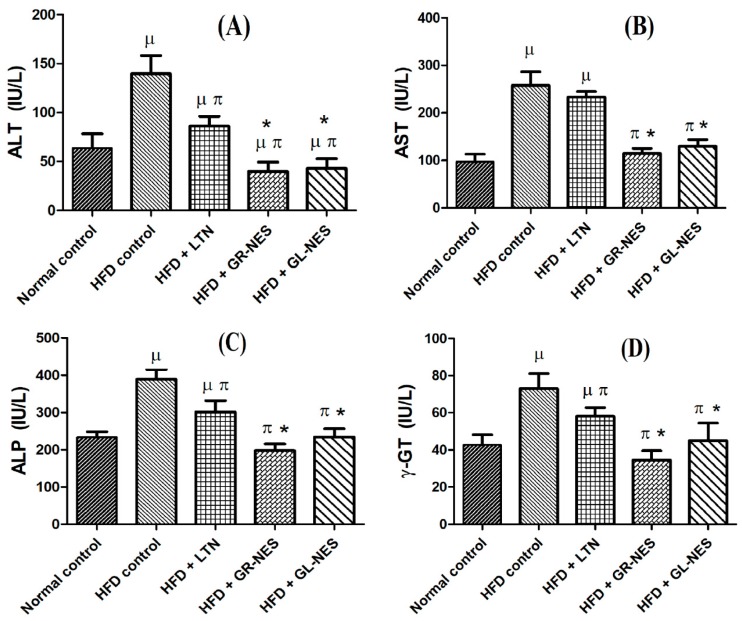
(**A**) Serum ALT; (**B**) Serum AST; (**C**) Serum ALP; (**D**) serum γ-GT levels of nanoformulations and pure LTN in hyperlipidemic rat models (*n* = 6) after 4 weeks of oral administration (dose = 30 mg/kg/d). µ indicates *p* < 0.05 vs. normal control; π indicates *p* < 0.05 vs. HFD control; * indicates *p* < 0.05 vs. HFD + LTN.

Group B kept on a high-fat diet appeared to have a drastic increase (277%) in plasma ALT (168 ± 18.69 IU/L), which was significantly greater (*p* < 0.05) than that of normal control group A (60.7 ± 14.76 IU/L; Figure 4A). The group treated with pure LTN suspension showed a 50% reduction (*p* < 0.05) in plasma ALT levels (84 ± 9.77 IU/L) as compared to those of group B, but these results were comparatively higher (i.e., 28% and 38% higher) than ALT levels of group D (60.7 ± 10.1 IU/L) and group E (52.9 ± 9.38 IU/L), respectively. Animals treated in groups D and E showed significantly reduced ALT levels (*p* < 0.05) in comparison to those of group B, indicating that animals of these groups recovered from NAFLD in a better way as compared to the other groups. These results are in accordance with a previous study [37].

#### 3.4.2. Aspartate Aminotransferase (AST or SGOT)

The serum AST level of group A was 98 ± 16.09 IU/L, while that of group B was 257 ± 28.75 IU/L. There was a highly significant difference between AST values of groups A and B (*p* < 0.001), indicating drastic effects of a high-fat diet on normal liver functions (Figure 4B). Among all treated groups (C, D, and E), groups D and E showed significant reductions (*p* < 0.05) in AST levels in comparison to group B. After completion of the experiment, their AST levels were 121 ± 10.05 and 143 ± 14.5 IU/L, respectively. Animals of group C did not display a significant reduction in AST levels when compared with group B, but these levels were significantly higher than those of groups D and E (*p* < 0.05).

#### 3.4.3. Alkaline Phosphatase (ALP)

Groups D and E were found to be most effectively protected after completion of the experiment. Both groups showed serum ALP levels after termination of the animal study at 196 ± 17.7 and 234 ± 22.15 IU/L, respectively. Their ALP levels seem to be in range of normal control group A (227 ± 15.05 IU/L). HFD group B showed a marked difference from group A (171% higher level) (Figure 4C). Four-week-long administration of GR and GL oil formulations showed respective 40% and 50% decreases in ALP levels as compared to group B. This proved the ameliorative effect of GR and GL oil formulations on normal functioning of liver enzymes.

#### 3.4.4. Gamma Glutamyl Transpeptidase (γ-GT)

Figure 4D displays serum γ-GT levels of all groups. It is evident that group B showed significantly (*p* < 0.05) higher levels of serum γ-GT as compared to group A, which was due to a high fat intake leading to hepatic damage. On the other hand, groups C, D, and E showed normalized values of serum γ-GT, indicating that treatment was beneficial for liver function. Among all treated groups, GR and GL oil nanoemulsomes exerted a comparatively better recovery as compared to LTN alone.

### 3.5. Renal Function Tests (RFTs)

Various serum biomarkers were measured to observe normal or abnormal renal functioning after consumption of a fat-rich diet. Serum creatinine, blood urea, BUN, serum proteins, albumin to globulin (A/G) ratio, and serum calcium were analyzed. Results show that group B was more prone to develop kidney disease or nephrotoxicity. Serum creatinine, blood urea, and BUN levels of group B were much higher (*p* < 0.05) at 143%, 116%, and 199%, respectively, than those of normal control group A, indicating establishment of nephrotoxic conditions and renal impairment in rats fed with a high-fat diet (Table 3). 

Blood urea levels of groups D and E showed respective 40% and 24% reductions (*p* < 0.05) when compared to those of HFD control group B. Differences between blood urea levels of groups B and C (6%) were not significant (*p* > 0.05). Blood urea levels of groups B and C were significantly different (*p* < 0.05) from those of normal control group A. Except for group C, serum creatinine levels of groups D and E illustrated significant decreases (*p* < 0.05) as compared to those of HFD group B (35% and 23% lower than group B, respectively).

BUN levels of groups D and E did not display significant differences (*p* > 0.05) with group A, while group C showed a significant difference (*p* < 0.05) with group A. There were significant differences (*p* < 0.05) in BUN levels of groups C, D, and E when compared with those of HFD group B. BUN levels of groups D and E reduced up to 66% and 51%, respectively, as compared to group B.

Serum proteins are taken as a direct measure of normal renal functionality. Results showed that serum total proteins, serum albumin, and A/G ratio of HFD group B were significantly different (*p* < 0.05) from that of group A (Table 4). Their values were 44%, 54%, and 17% lower, respectively, as compared to normal diet group A. Whereas, serum globulin of HFD group B was 304% greater than that of group A. These results demonstrate the prevalence of kidney disease in hyperlipidemic rats.

Groups treated with the LTN suspension and drug-loaded GR and GL oil nanoemulsomes demonstrated improved kidney functions by the normalized values of these biomarkers, but they showed significant differences (*p* < 0.05) with HFD group B. Group C registered an increase of 194%, 137%, and 374% in total proteins, serum albumin, and A/G ratio, respectively, while a decrease of 36% was seen in serum globulin levels as compared to group B.

Groups D and E also manifested amelioration of 208% and 180% in total proteins, 162% and 131% in serum albumin, and 414% and 319% in the A/G ratio, respectively, as compared to HFD group B. Furthermore, there were decreases of 39% and 33% in serum globulin levels of groups D and E, respectively, as compared to group B.

### 3.6. Histopathological Examination

**Figure 5:**
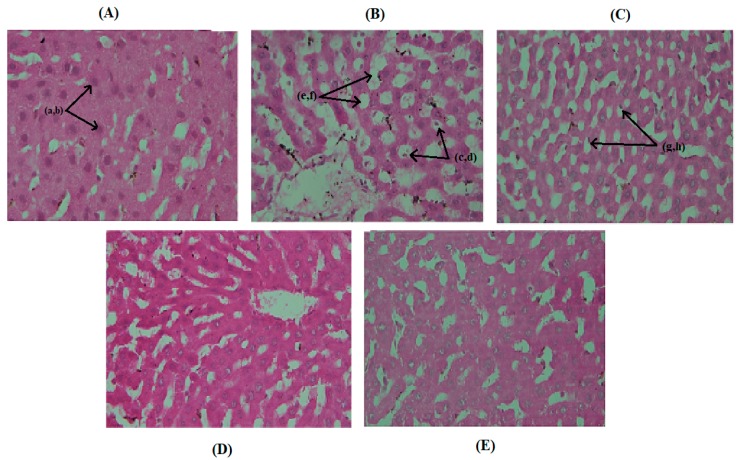
(**A**) Liver section of a rat of from the normal diet group, arrows (a, b) indicate localized nuclei with well-established cytoplasm; (**B**) liver section of a rat kept on a high-fat diet, arrows (c, d, e, and f) demonstrate bulged out, fat-filled large sacs (ballooning) and major displacements of cell nuclei; (**C**) liver sections of LTN-treated rats indicating mildly fat-filled or microvesicular structures with displaced cell nuclei (arrows g, h); (**D**) liver section from the GL oil LTN-loaded nanoemulsome treatment group claiming no vesicular ballooning or fat accumulation; (**E**) liver section from the GR oil LTN-loaded nanoemulsome treatment group displaying cytoplasmic preservation with no fat deposition but minute displacement of cell nuclei.

Histopathological results of liver sections of normal control diet group A (Figure 5A) indicated normal hepatocytes with prominent nuclei and preserved cytoplasmic structures without the occurrence of ballooning or necrotic effects. Nuclei in these hepatocytes were not displaced, and cellular structures were well maintained. H&E sections of animal livers maintained on a hyper fat diet (group B) indicated swollen globular sacs (ballooning effect) with major nuclei displacements and hepatocytic degenerative effects (Figure 5B), demonstrating the accumulation of excess fatty acids. Liver sections of group C, treated with LTN suspension, also claimed nuclei displacements, but there were mild ballooning degenerative effects (Figure 5C) as compared to group B on liver parenchyma cells, indicating ameliorative effects of LTN towards damaged liver cells but not yet fully recovered. Both GL and GR oil LTN-loaded nanoemulsomes imparted magnificent curative results, as liver sections of these groups (D and E) displayed a well-organized structure of hepatocytes along with established cytoplasmic material (Figure 5D,E).

**Figure 6:**
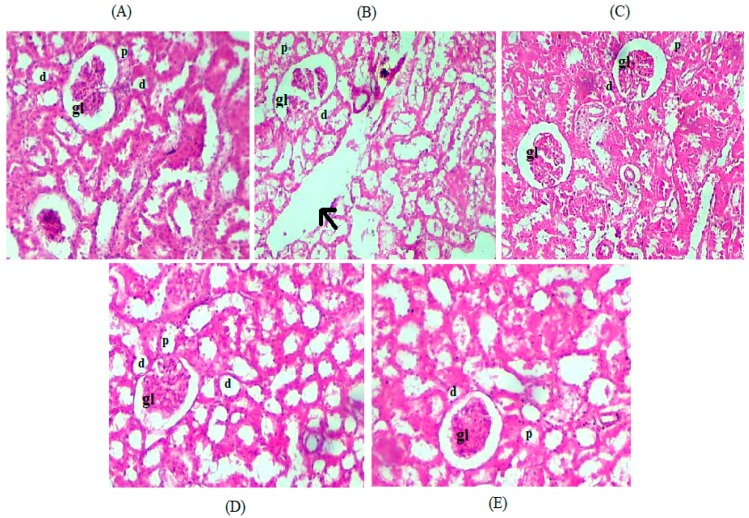
(**A**) Kidney section from the normal control group. (**B**) Kidney section from the high-fat diet group. Arrow indicates enlarged blood vessels. (**C**) Kidney section of the LTN-treated group. (**D**,**E**) Kidney sections of animals treated with garlic and ginger oil LTN-loaded NESs, respectively. Black arrows indicate dilated blood vessels. d; distal convoluted tubule. p; proximal convoluted tubules. gl; glomerulus.

Histopathology of H&E stained kidney sections of experimental rats from control group A (Figure 6A) demonstrated normal kidney structures, as the glomeruli of these rats were found to be in proper shape and in preserved form, with no dilation of interstitial blood vessels and renal tubules, indicating normal functioning of kidneys in these rats. While in the case of group B (HFD group), H&E stained kidney sections showed degenerated glomerular structures (dilated glomerular capillaries) along with dilated blood vessels and unpreserved renal tubules (distal and proximal tubules) with enlarged lumen (Figure 6B). Group C, which was treated with pure LTN suspension, displayed improved glomeruli, but renal tubules remained unpreserved due to the effect of the high-fat diet (Figure 6C). Glomerular capillaries were also dilated, but to a lesser degree, as compared to those in group B. Groups D and E showed that glomerular structures in both groups were in a far better position and were comparable to that in normal control group A (Figure 6D,E). Both groups demonstrated a well-preserved renal tubular system with no blood vessel dilations. 

## 4. Discussion

### 4.1. Body Weight of Animals

Among animal obesity models, rats that are fed a continuous hyper fat diet are usually preferred because they develop NAFLD by long-term consumption of a high-fat diet leading to a hyper body weight, increased lipid levels, boosted arterial deposition of cholesterol, hepatic steatosis, and dyslipidemia [38]. In our study, group A sustained normal body weight throughout experiment, while hyper fat diet consumption in rats (group B) resulted in tremendous weight gain of about 200% as a consequence of high energy intake and mass building, which is attributed to saturated fat deposition in different body fat pads [39]. This high level gain in body weight stipulates two mechanisms of adiposity: increase in cell number (hyperplasticity) and increase in cell size (hypertrophy) [40]. 

Groups C, D, and E also showed similar patterns as group B until week 4, when treatment was started. After the first week of treatment, groups treated with drug-loaded GR and GL oil nanoemulsomes showed decreases in percent body weight, which were attributed to the synergistic antiobesity effects of LTN and active constituents present in these oils. Group D treated with GR oil LTN-loaded nanoemulsomes was significantly different (*p* < 0.05) from the hyper fat diet group B in terms of body weight percent and substantially lowered the body weights of these animals. This is due to the presence of 6-gingerol, which might have caused hypophagic effects in obese animals [41]. 

GR also produces hurdles in absorption of dietary fats by retarding fat hydrolysis, which results in loss of adipose tissue weight [42]. GR aqueous and alcoholic extracts have been shown to produce effective antiobesity effects in animals [42]. Likewise, GL oil drug-loaded nanoemulsome treatment group E also showed reduction in body weight percent after initiation of treatment. This is because of DADS, a highly active organosulfur compound of GL, which helps in lowering diet-induced body weight and adipose tissue weight as well [43].

### 4.2. Lipid Profile

Globally, dyslipidemia is known to be an established cardiovascular risk factor, and, if left uncured, its persistence causes fatalities like atherosclerosis and chronic heart disease [44]. Results obtained from animal serum data analyses provide satisfactory evidence of ameliorating biochemical parameters of the lipid profile (i.e., TC, LDL-C, vLDL-C, HDL-C, TG, and AI). Lower serum TC levels in groups treated with drug-loaded nanoemulsome formulations indicate enhanced and prolonged effects of LTN because of their raised carrying capacity of hydrophobic moieties [45]. It is evident from the results that there was a significant reduction (*p* < 0.05) in serum TC levels of groups D and E as compared to those of group B and the group treated with pure LTN suspension, which indicates significant effects on the cholesterol metabolic sites by nanoemulsomes. 

There are number of reported studies in which these oils have markedly reduced serum TC levels in hypercholesterolemic animal models [4,46]. A possible explanation for the greatly lowered serum TC levels of groups D and E may be the synergistic effects of LTN combined with these essential oils. GR essential oil contains numerous polyphenolic and other volatile constituents like geranaiol, citral, gingerol, zingeberene, β-funebrene, citronellyl n-butyrate, α-Pinene, and camphene, which have proven to be effective in lowering serum TC levels in hypercholesterolemic animals [47]. Decreased levels of TC of group D might have originated from HMG-Co-A reductase inhibitory effects of one of the components of GR (i.e., aframodial [48]).

10-dehydrogingerdione, another component isolated from GR, has reportedly decreased TC levels of hypercholesterolemic rats by inhibiting cholesteryl ester transfer proteins (CETP), which are proteins that help reverse transport of cholesteryl ester from HDL-C towards triglyceride-rich lipoproteins (LDL-C and vLDL-C) [49]. This component of GR has also been shown to raise HDL-C levels [50]. Previously, GR and GL oil components have been shown to be effective in decreasing TC levels and improving the lipid profile in hyperlipidemic rat models by retarding the activities of several enzymes that are directly involved in cholesterol synthesis like SREBP1-c and ACC [4,12].

GR constituents also alleviate TC levels via another reported mechanism of upgrading hepatic cholesterol 7α-hydroxylase activity. This is a rate-limiting enzyme in the conversion of serum free cholesterol to bile acids and downregulating *HMGR* genes (responsible for encoding rate-limiting enzymes in cholesterol biosynthesis), hence lowering serum TC and the risk of coronary heart disease [48,51]. Remarkably reduced levels (*p* < 0.05) of vLDL-C, LDL-C, and triglycerides and elevated levels of HDL-C can be explained by the presence of niacin in GR extracts. Niacin helps to clear vLDL-C, attenuate serum TG levels, and boost hepatic LDL-C uptake and downregulate its oxidation process as well [52]. Gingerol, another component of GR oil, tends to amend the lipid profile by boosting HDL-C levels and helps reduce absorption of plasma and tissue cholesterol via inhibition of pancreatic lipase [46].

Group E treated with nanoemulsomes carrying GL oil as liquid lipid also showed markedly reduced (*p* < 0.05) levels of TC and a revamped lipid profile of hypercholesterolemic rat models as compared to group B and the LTN-treated group. Organosulfur compounds in GL tend to refine antioxidant properties by increasing glutathione levels and antioxidant enzyme activity as well as relieving lipid peroxidation [53,54]. Certain constituents (allicin, DADS, and allyl mercaptan) of GL inhibit biosynthesis of cholesterol directly by inhibiting HMG-Co-A reductase [55]. 

Allicin and DADS have shown to lower levels of free fatty acids (FFAs), and body weight as well, owing to reduced adipose tissues over the continuous administration of drug-loaded GL oil nanovesicular lipoidal formulations. This decreased weight after the fourth week of study could be attributed to the anti-hyperlipidemic and antiobesity effects of GL, and it has a direct link in reducing LDL-C, vLDL-C, and TG levels [43]. In a study, a commercially available combination (PENNEL^®^) of GL oil and dimethyl-4,4-dimethoxy-5,6,5,6-dimethylenedioxybiphenyl-2,2-dicarboxylate (DDB) significantly reduced serum TG levels [56]. Similar to GR oil, GL oil has also been reported in eliminating atherosclerotic effects and lowering AI by inhibiting CETP and improving serum HDL-C levels [57].

GL oil manipulates biosynthesis of cholesterol and bad lipids by directly inhibiting the number of enzymatic processes, which involve inhibition of fatty acid synthase, glucose-6 phosphate dehydrogenase (G-6 PDH), and malic enzyme as well. Therefore, in our study, GL substantially assisted LTN in directly blocking HMG-Co-A reductase and showing synergy via reported mechanisms [58]. GL oil LTN-loaded nanoemulsomes showed significantly reduced TC levels, while HDL-C levels were boosted. This can be attributed to the cholesterol excretion mechanism of GL, which manifests in elimination of acidic and neutral steroids, and also because of reduced vLDL-C fractions and HDL-C-induced removal of cholesterol from arterial tissues [57]. Since GL oil contains allicin, it exerts antioxidant effects towards LDL-C, making it possible to alleviate the risk of atherosclerosis [59].

Anti-hyperlipidemic agents work by either lowering the bad lipids (TG, LDL-C, and vLDL-C) or by increasing the good lipids (HDL-C) in plasma, or a combination of both. Our study demonstrated that both GL and GR decreased the bad lipids and increased the good lipids at the same time. However, GR was superior in lowering the bad lipids than GL, and GL was quite better in improving the good lipids as compared to GR. With this in mind, AI, a measure of the overall atherogenic potential, is the ratio of bad lipids to good lipids. Thus, AI results of this study reveal that GR-NES was comparatively good at lowering the risk of atherogenesis than the GL-NES. The risk of coronary heart disease increases with an increased presence of atherosclerosis, which manifests as depositions of bad cholesterol and fats on the walls of arteries. Hence, AI is usually considered as a stand-alone index to evaluate cardiovascular risk factors when all other parameters of the serum lipid profile are ruled out or insufficient in providing necessary information [60]. LTN has proved to be an effective agent in improving the lipid profile in hypercholesterolemic or dyslipidemic animal models, hence leading to reduced risk of AI [61]. In our study, LTN along with GR and GL oils (drug-loaded nanoemulsomes) provided the best treatment in scaling down AI of animals kept on a high-fat diet. This is regarded as the direct action of LTN in improving the lipid profile of hyperlipidemic rats in synergy with parallel effects of GR and GL oil constituents, hence, lowering the risk of AI by decreasing LDL-C and vLDL-C while improving HDL-C levels [12]. In this study, we also considered the TG/HDL-C ratio as the surrogate indicator for insulin resistance as suggested in recent literature [33]. GR-NES and GL-NES were much better than LTN alone in reversing insulin resistance. Nanosomal formulations reduced insulin resistance in hyperlipidemic rats to the same level as that of the rats fed on a normal or control diet. Our findings comply well with the reported anti-insulin resistant properties of both GR [62] and GL [63].

### 4.3. Liver Function Analyses (LFTs)

The liver is known to be the main site for biosynthesis and metabolism of number of molecules. Any damage to it leads to abnormalities in normal physiological functions of body. Excess intake of a diet rich in fatty acids deteriorates the normal functioning of hepatocytes. High-fat diet induces changes in the normal physiology of hepatocytes, by depositing fat inside them, as it exerts pressure on cell linings and makes them leaky [37]. Since alkaline phosphatase (ALP) is a membrane-bound enzyme and found to be in abundance in hepatocyte cell membranes, it tends to escape cells once they become leaky, therefore ALP levels are high in animals that have been fed a high-fat diet [64]. While AST and ALT are abundantly found enzymes in kidney and liver only, their high serum concentrations occur when cellular gateways are open or are made open by physiological or toxicological changes in normal cell functions [65]. This evidence helps understand our findings of the high serum AST and AST levels in the HFD group thatexcess feeding of a fat-rich diet led to ample amounts of saturated fat inside the hepatocytes, acquiring enormous space, and making cell membranes leaky by pushing cellular material to the peripheries. High serum concentrations of these localized enzymes illustrate HFD induced insult and damage to cell membranes [37].

Hence, administration of LTN-loaded GR and GL oil lipoidal nanovesicular systems boosted the healing process of HFD-ruptured cell membranes and helped lower serum concentrations of AST, ALT, GGT, and ALP. Dietary lipids have shown to boost the regulation of membrane lipid composition, which ultimately helps in controlling the activity of membrane proteins like ALP [66]. GR has improved the serum profiles of AST, ALT, GGT, and ALP by healing hepatocyte membranes, as GR is known to be a highly hepatoprotective neutraceutical agent. GR has previously shown marked protection of liver cells from injuries with an accelerated healing process by uplifting the activity of the antioxidant enzyme (superoxide dismutase (SOD)) and by slowing the lipid peroxidation processes [67]. The antioxidant activity of GR oil resembles that of vitamin C in lowering lipid peroxidation by impacting serum catalase and SOD enzyme concentrations [68]. GR oil nanoemulsomes also significantly (*p* < 0.05) reduced serum GGT levels, owing to its antioxidant ability [16].

GR extracts have also been effective in regenerating parenchyma cells, protecting against membrane fluidity and fragility, and promoting rapid normalization [69]. Our results of lower AST, ALT, and ALP activities in improving cellular health and minimizing leakage from hepatocytes upon administration of GR oil nanoemulsomes are also in accordance with previous studies [70]. Two grams of GR powder, when administered for about 12 weeks, led to significantly reduced serum levels of ALT in NAFLD patients [71]. Atorvastatin and GR extract were shown to promote recovery of cell membranes from leakage defects and downregulated the activities of AST, ALT, GGT, and ALP by inhibiting their escape from ruptured hepatocytes [72].

In a recent study, GL oil significantly alleviated AST, ALT, GGT, and ALP concentrations to satisfactory levels by providing recovery to damaged cell linings of liver cells and because of its antioxidant activities (i.e., uplifting activity of SOD enzyme [73]). Hepatotoxicity induced by paracetamol was cured, and hepatocytes were protected from injury by administration of GL extract. This finding was attributed to GL oil, as it contains S-allyl cysteine (SAC) and S-allylmercapto cysteine (SAMC). These two constituents of GL oil, especially SEMC, tend to impart hepatoprotective properties to the injured cell membranes [22]. DAS, being an important and major component of GL oil, has shown revamped results in ameliorating stress-induced or alcohol-induced fatty liver disorders. It directly inhibits the P450 2E1 enzyme, which is involved in lipid peroxidation intoxication of a number of metabolic toxic products in such abusive conditions that cause further damage to cell physiology.

Previous research works showed that DAS has successfully inhibited or downregulated the activity of the CYP2E1 enzyme upon administration of GL oil in hepatic disease conditions [74]. Enzyme CYP2E1 is actively involved in the metabolism of a number of small toxic molecules like CCL_4_. Inhibition of this enzyme by GL indicated improved hepatocyte histopathology and also decreased serum levels of AST, ALT, GGT, and ALP. This pharmacological action of GL oil is attributed to the presence of highly antioxidant organosulfur compounds found in it (AS, DAS, and DADS) [56]. Likewise, citral (a component of GR) has also been revealed to reverse CYP2E1 activity in a dose-dependent manner, where moderate to higher doses of citral and GR essential oil substantially turned off this enzyme’s activity [4].

### 4.4. Renal Function Tests (RFTs)

Chronic dietary lipid intake leads to excess deposition of saturated fats inside the cells of various organs. Lipids are deposited in the kidney at a high level, leading to significant alterations in renal subcellular structures (renal cortex) [75]. Enormous adiposity leads towards dilation of large localized blood vessels, and subcapsular adipocyte accumulation causes glomerular atrophy and necrosis, resulting in poor filtration of a number molecules and, consequently, raising their plasma levels [76]. Vasculature abnormalities and nephropathy, owing to a high intake of saturated fatty acids, has also been reported [77]. Hypercholesterolemia, hypertriglyceridemia, low levels of HDL-C, and high concentrations of apolipoprotein-B are known as major risk factors in the development of chronic kidney disease (CKD). Moreover, in the presence of these abnormalities, kidney disease progresses more rapidly [78]. Such nephrotoxic and abnormal conditions lead to malfunctioning of kidneys and cause an imbalance in concentrations of small molecules and electrolytes inside and outside the cells [79].

Elevated levels of blood urea and creatinine are direct indicators of renal injury due to enhanced xanthine oxidase, TG, and cholesterol levels, and this indicates uplifted lipid peroxidation in hypercholesterolemic rats [9,80]. It is evident from the results that group B, which was kept on a high-fat diet throughout the experiment, showed comparatively higher levels of serum creatinine, blood urea, serum total calcium, decreased serum total proteins, and albumin-globulin (A/G) ratio. Whereas, the levels of these biochemical parameters in groups treated with LTN-loaded GR and GL oil nanoemulsomes (groups D and E) were quite lower compared to the HFD group. The reason behind this is the direct action of these natural therapeutic agents in remediation of injured cell lines and damaged organs.

GR aqueous and alcoholic extracts have been shown to normalize levels of these biomarkers and heal the damaged site [81]. GR contains polyphenols and flavonoids, which provide antioxidant and nephroprotective effects and help regulate the normal functioning of nephrons [82]. This prevents elevations in blood urea and serum creatinine levels and reductions in serum proteins and albumin leakage into urine (increase in the A/G ratio). Hence, it normalized the levels of these biomarkers as compared to HFD group B. GR is widely known as a blood cleaner when it comes to elevated levels of blood urea and serum creatinine; hence, it keeps normal concentrations of these biological compounds by eliminating them when their concentration goes higher in diseased conditions [83].

Gingerol has been designated as a potent chemical in downregulating inflammatory gene expressions at the site of injury, and it protects against oxidative stress by dwindling the concentration of nitric oxide (NO) [34,84]. HFD group B showed reduced levels of serum proteins and the A/G ratio, indicating greater removal of proteins (total proteins and albumin) into urine as a result of leaky glomerular linings and tubules [80]. Group D showed reduced leakage, normal levels of the proteins, and a comparatively higher A/G ratio in serum, owing to GR’s amending properties on injured organs [81]. 

Both GL and GR oil nanoemulsomes caused reductions in creatinine levels, as the body weights of these groups decreased. This evidence is in accordance with a previous report [85]. Lipids play a major role in the establishment of glomerular disease, and all lipid-lowering agents are involved in protecting the kidney from getting injured. LTN indirectly protects the kidneys from nephritic conditions by ameliorating glomerulosclerosis and by inhibiting ingestion of lipoproteins in mesangial cells and preventing binding of these lipids to glomerular epithelial cell linings [25,86]. Besides, statins have also shown their direct effect in retarding progression of CKD via a number of mechanisms [23,87]. Simvastatin and fluvastatin have directly imparted renoprotective effects in clinical trials by combating dyslipidemia and reversing proteinuria [88]. This evidence seconds our findings on the synergistic effects of LTN with GR or GL oils in regulating renal functions.

Gulaz et al. found out that GL oil protects kidneys from damage and does not allow any alterations in the sizes of glomeruli in rats induced with acetaminophen nephrotoxicity. GL oil improved the functioning of glomeruli in comparison to diseased ones. Animal urea, A/G ratio, and serum creatinine levels were also found to be in normal ranges as compared to diseased animals, owing to the antioxidant properties of organosulfur compounds of GL oil [89]. GL aqueous extract has helped in the recovery from glomerular ischemia and free radical induced oxidative stress and has also improved BUN, blood urea, and serum creatinine levels [90]. GL has also been reported to restore serum levels of biomarkers of normal renal functions by protecting glomeruli from reactive oxygen species (ROS) by boosting levels of antioxidant enzymes [91]. DADS has also been widely approved for its antioxidant activity and beneficial role in protecting the kidney from nephrotoxicity [21].

### 4.5. Histopathology

Obesity, hypercholesterolemia or hyperlipidemia, and genetically altered fatty acid metabolism are considered to the major causes of fatty liver disease (FLD) [92]. If left untreated, these conditions may lead to the occurrence of liver cirrhosis, liver cancer, and steatohepatitis [93]. In this study, liver sections of animals fed on a hyper fat diet (Figure 5B) showed excess accumulation of lipids (lipid-filled macrovesicular structures), indicating prevalence of hepatic steatosis and hepatic necrosis (excessive fat deposition in hepatocytes appearing as a rounded ring with a swelled vacuole and displaced cell nuclei) [94]. This evidence proves the occurrence of fatty liver disease in hypercholesterolemic rats. Liver sections of group C animals treated with LTN suspension alone (Figure 5C) exerted slight curative effects in reducing fat accumulation, but they also showed fat-filled sacs of reduced sizes as compared to ones that appeared in the high-fat diet group B. Generally, hepatosteatosis takes place because of the presence of microvesicular small fat droplets that are responsible for filling hepatocyte cytoplasm without a central location of the nucleus [95]. Liver sections treated with GL and GR oil LTN-loaded nanoemulsomes (groups D and E) showed substantially improved histological features with preserved cytoplasmic material and demonstrated no existence of fat-filled large or small vesicles. Nuclei in these sections (Figure 5D,E) look active and larger (vesicular) with a normal appearance of blood sinusoids, which describes the restorative effects of LTN on hepatocytes in synergy with GR and GL oils [70]. 

According to a recent report, obesity bears a direct link in establishing metabolic syndrome in effected individuals, which leads to CKD and damages the functional units of kidneys (glomeruli and renal tubules). It manifests because of ROS produced in response to hyperlipidemic stress and kidney injuries due to high fat accumulation [96]. Group B illustrated enlarged renal blood vessels as well as degenerated and dilated glomerular capillaries and renal tubules (Figure 6B) associated with fat accumulation [76]. Group C (Figure 6C) displayed improved morphology of glomerular capillaries as compared to the HFD group. This improvement is attributed to the direct renoprotective effect of LTN [25]. Histopathological findings indicate improved and preserved kidney structures for NES-treated groups. This can be attributed to the renoprotective effects of active compounds (flavonoids steroids and alkaloids) found in ginger that help mop up free radical generation and protect the kidney from injury [97]. The renoprotective activity of garlic’s sulfur containing compounds (DAS and allicin), which tend to inhibit the production and activity of ROS produced in response to hypercholesterolemic renal injuries [98], might also explain the performance of GL-NES.

## 5. Conclusions

Data of the current study demonstrate that co-encapsulation of LTN with natural therapeutic oils (GR or GL) in a lipoidal nanovesicular delivery system results in significant improvement in the therapeutic efficacy of standard anti-hyperlipidemic drugs. This improvement in the anti-hyperlipidemic profile is suggested to be due to the presence of multiple active components. Exploiting the curative effects of such oils, along with a pharmaceutical active moiety, not only allows better control of the diseased state but also provides protection to the key organs. Hence, the synergistic activity of nutraceutical and pharmaceutical ingredients leads to a successful therapy with a quick and efficient recovery. This could lead to a reduction in the dose of the given drug, consequently decreasing the drug-associated side effects. Hence, we conclude that GR and GL oil LTN-loaded nanoemulsomes could be an effective strategy against hyperlipidemia and associated complications, which involves the use of naturally occurring therapeutic constituents in tandem with the drug to improve the overall therapy and, hence, patient compliance [99].

## Figures and Tables

**Table 1:** Composition of lovastatin (LTN)-loaded nanoemulsomes prepared by thin layer hydration.

Formulation Code	Stearic Acid (mg)	Garlic (GL) Oil (mg)	Ginger (GR) Oil (mg)	Phospholipon 90G (mg)	LTN (mg)
NES-GL 1:2	27	53	--	330	20
NES-GR 1:2	27	--	53	330	20

**Table 2:** Particle size, zeta potential, and polydispersity index (PDI) of formed nanoemulsomes.

Formulation Code	Particle Size (nm)	Zeta Potential (mV)	PDI
Garlic Nanoemulsome (NES-GL) 1:2	240 ± 5.5	−24 ± 2.4	0.25 ± 0.08
Ginger Nanoemulsome (NES-GR) 1:2	120 ± 4.2	−20 ± 4.8	0.126 ± 0.06

**Table 3:** Effect of nanoformulations and pure LTN on serum biomarkers for kidney functions in hyperlipidemic rat models (*n* = 6) after 4 weeks of oral administration (dose = 30 mg/kg/day).

Groups	Serum Creatinine (mg/dL)	Blood Urea (mg/dL)	BUN (mg/dL)
Normal control (A)	0.32 ± 0.04	46 ± 4.50	21.5 ± 4.28
HFD control (B)	0.46 ± 0.07 *	51 ± 5.31 *	42.9 ± 8.03 *
HFD + LTN (C)	0.41 ± 0.04	48 ± 3.41 * # µ	30.3 ± 4.75 * #
HFD + GR-NES (D)	0.30 ± 0.03 #	31 ± 2.73 * # µ	14.7 ± 2.55 # µ
HFD + GL-NES (E)	0.34 ± 0.03 #	38 ± 3.71 * #	21.2 ± 3.03 #

* indicates *p* < 0.05 vs. normal control; **#** indicates *p* < 0.05 vs. HFD control; µ indicates *p* < 0.05 vs. HFD + LTN.

**Table 4:** Effect of nanoformulations and pure LTN on serum biomarkers for kidney functions in hyperlipidemic rat models (*n* = 6) after 4 weeks of oral administration (dose = 30 mg/kg/d).

Groups	Serum Protein (g/dL)	Serum Albumin (g/dL)	Serum Globulin (g/dL)	A/G Ratio
Normal control (A)	7.6 ± 0.76	4.92 ± 0.63	2.62 ± 0.40	1.8 ± 0.20
HFD control (B)	3.4 ± 0.93 *	2.67 ± 0.46 *	7.98 ± 0.54 *	0.33 ± 0.07 *
HFD + LTN (C)	6.6 ± 0.65 #	3.67 ± 0.38 #	2.93 ± 0.45 #	1.25 ± 0.16 #
HFD + GR-NES (D)	7.1 ± 0.58 #	4.34 ± 0.50 #	3.13 ± 0.60 #	1.38 ± 0.28 #
HFD + GL-NES (E)	6.14 ± 0.46 #	3.5 ± 0.68 #	2.64 ± 0.42 #	1.32 ± 0.20 #

* indicates *p* < 0.05 vs. normal control; # indicates *p* < 0.05 vs. HFD control.
